# First crystal structure of an endo-levanase – the BT1760 from a human gut commensal *Bacteroides thetaiotaomicron*

**DOI:** 10.1038/s41598-019-44785-0

**Published:** 2019-06-11

**Authors:** Karin Ernits, Priit Eek, Tiit Lukk, Triinu Visnapuu, Tiina Alamäe

**Affiliations:** 1 Department of Genetics, Institute of Molecular and Cell Biology, University of Tartu, Riia 23, 51010 Tartu, Estonia; 2 Department of Chemistry and Biotechnology, Tallinn University of Technology, Akadeemia tee 15, 12618 Tallinn, Estonia

**Keywords:** Hydrolases, X-ray crystallography, Prokaryote, Polysaccharides

## Abstract

The endo-levanase BT1760 of a human gut commensal *Bacteroides thetaiotaomicron* randomly cuts a β-2,6-linked fructan, levan, into fructo-oligosaccharides providing a prebiotic substrate for gut microbiota. Here we introduce the crystal structure of BT1760 at resolution of 1.65 Å. The fold of the enzyme is typical for GH32 family proteins: a catalytic N-terminal five-bladed β-propeller connected with a C-terminal β-sandwich domain. The levantetraose-bound structure of catalytically inactive mutant E221A at 1.90-Å resolution reveals differences in substrate binding between the endo-acting fructanases. A shallow substrate-binding pocket of the endo-inulinase INU2 of *Aspergillus ficuum* binds at least three fructose residues at its flat bottom. In the levantetraose-soaked crystal of the endo-levanase E221A mutant the ligand was bent into the pond-like substrate pocket with its fructose residues making contacts at −3, −2, −1 and + 1 subsites residing at several pocket depths. Binding of levantetraose to the β-sandwich domain was not detected. The N- and C-terminal modules of BT1760 did not bind levan if expressed separately, the catalytic domain lost its activity and both modules tended to precipitate. We gather that endo-levanase BT1760 requires both domains for correct folding, solubility and stability of the protein.

## Introduction

Gut microbiota of healthy adults has two dominating bacterial phyla: the Firmicutes (Gram-positive, many genera) and the Bacteroidetes (Gram-negative: *Bacteroides*, *Parabacteroides*, *Prevotella*, *Alistipes* and others)^1^. A unique feature of Bacteroidetes is their ability to degrade and ferment diverse polysaccharides that allows feeding on dietary fibre – poly- and oligosaccharides not digested by human enzymes^1,2^. These bacteria degrade for example resistant starch, pectin, galactomannan, glucomannan, arabinogalactan, alginate, laminarin, xylan, β-glucan, rhamnogalactan, cellulose and levan^3–5^. Polysaccharide degradation abilities of Bacteroidetes are encoded in specific polysaccharide utilization loci (PULs). As a rule, the PULs also encode surface-bound endo-acting enzymes that initiate sugar polymer degradation^6^. In the fructan PUL, this role is fulfilled by the endo-levanase encoded by *BT1760*^3^. Levans, β-2,6-linked fructose polymers, have currently gained attention as polysaccharides with many medical, food-related and technological applications^7–10^. Notably, β-2,6-linked fructo-oligosaccharides (L-FOS) resulting from levan hydrolysis were shown having even higher prebiotic effect on probiotic gut bacteria than commercial β-2,1-linked I-FOS obtained from inulin degradation^11–14^. As L-FOS are not commercially produced, their prebiotic effects are yet poorly studied. We have studied biochemical properties of endo-levanase BT1760 and shown its very high catalytic activity and ability to produce L-FOS from various levans^15^. Thus, BT1760 may have a biotechnological application in large-scale production of L-FOS for the studies of their physiological effects. As levans are very large and usually branched molecules of molecular weight reaching megadaltons^7,15^, this endo-acting enzyme offers great interest from the aspect of structural biology of enzymes.

Considering endo-acting fructanases, the crystal structure is available only for the endo-inulinase INU2 of a filamentous fungus *Aspergillus ficuum*^16^. With regard to levanases, only one structure was available during our studies: the structure of the C-terminal β-sandwich domain of the *Bacillus subtilis* exo-levanase SacC, a founding member of the CBM66 family of carbohydrate binding modules (CBMs)^17^.

Here we introduce the first crystal structures of the *B. thetaiotaomicron* endo-levanase BT1760 (EC 3.2.1.65). The structure of the catalytically active protein was resolved at 1.65 Å, and of its non-catalytic E221A mutant complexed with levantetraose at 1.90 Å. As typical for GH32 family enzymes, the BT1760 comprises an N-terminal five-bladed β-propeller catalytic domain and a tightly packed C-terminal β-sandwich module linked to it through a short helix. The substrate-binding pockets of the two endo-acting fructanases INU2 and BT1760 were shown to differ in shape and ligand binding mode. Previously, the C-terminal domain of BT1760 was characterized as a domain of unknown function (DUF4975)^15^. In the light of our results, we suggest that this domain, although structurally similar to carbohydrate binding modules (CBMs), is rather needed for folding, solubility and stability of the whole protein.

## Results

### Endo-levanase structure determination

The 508 aa-construct of endo-levanase BT1760 with C-terminal His_x6_-tag crystallized readily and yielded large (shortest dimension ~100 μm) rod-shaped crystals. This facilitated initial experiments on an in-house diffractometer with a sealed tube Cu-anode X-ray source. Diffraction data was collected to 2.0-Å resolution, however, our efforts to solve the structure by molecular replacement (MR) yielded indefinite results. The high quality of the data, the number of S atoms in the protein, and high crystal solvent content (about 60%) encouraged us to attempt sulphur-based single-wavelength anomalous dispersion (S-SAD) phasing. Multiple sweeps were collected from a single crystal to increase average redundancy to about 70. The merged data contained useful anomalous signal (correlation between half-dataset anomalous differences over 30%) to 4.0 Å. While the attempts to resolve the phases using only SAD data failed, we were able to solve the structure with phenix.AutoSol in space group I222 by additionally providing an ambiguous MR solution as a partial model, or in other words, by utilizing an MR-SAD approach. A native 1.65-Å dataset was subsequently used to further refine the model. Data collection and refinement statistics are available in Supplementary Table S1. The final model of BT1760 (PDB: 6R3R) contains 492 residues, 96.3% of which are in the favoured region of the Ramachandran plot and there are no outliers. In addition to the genetically removed signal peptide, the model is missing 14 residues from the N-terminus and 2 C-terminal His residues of the His_x6_-tag due to crystal disorder.

In crystallization trials, ZnCl_2_ emerged as an essential additive. Once the crystal structure was solved, the reason for that became evident: the protein crystallized as a 1:1 complex with Zn^2+^. The ion was tetragonally coordinated by His26, His384, His503 and His506, the latter two belonging to the His_x6_-tag, while His506 provided by the neighbouring symmetry-related molecule. Thus, the formed crystal actually comprises pairs of endo-levanase monomers that are linked by two closely situated Zn^2+^ coordination spheres, even though the asymmetric unit contains only one protein chain.

The inactive E221A mutant was crystallized under analogous conditions, albeit at lower protein and precipitant concentrations (see Materials and Methods for details). A native dataset to 1.90-Å resolution was collected from a crystal that was soaked overnight with levantetraose (L-FOS, FFFF), a levan degradation intermediate that can be further cleaved by the endo-levanase. The wild-type and mutant crystals were nearly isomorphous, the latter having the unit cell larger by 0.5–3 Å in each dimension. The refined model of E221A mutant (PDB: 6R3U) has 95.3% of residues in the Ramachandran favoured region, no outliers, and is missing 13 residues from its N-terminus similarly to the wild-type model. No pronounced differences between the two protein structures (r.m.s.d. across all Cα pairs is 0.25 Å) were detected except for the active site and His_x6_-tag. Unlike the wild-type structure, the electron density map of the E221A mutant was extremely difficult to interpret in the tag region. Several backbone conformations of the C-terminal Tyr502-His508 were detected in the crystal structure, possibly due to suboptimal protein:Zn^2+^ ratio, which in combination rendered each other indistinguishable. The model depicts only one plausible conformation of many, hence unresolved blobs remain in the *mF*_*o*_*-DF*_*c*_ difference map in that region. In the active site, Gln239 is partially found in an alternative conformation filling the void introduced by the E221A mutation. No other rotameric shifts were detected, so the integrity of the binding site was retained.

### Overall fold of the endo-levanase

**Figure 1:**
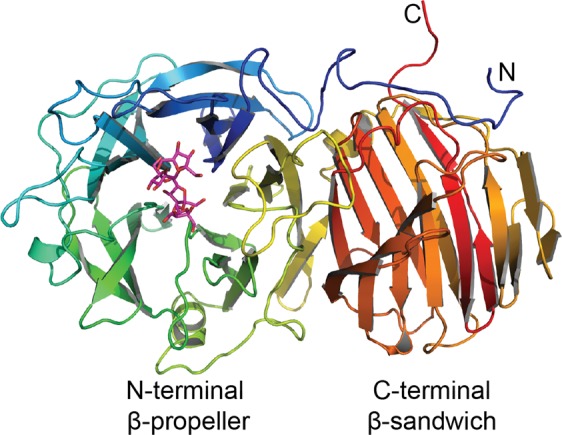
A ligand-bound structure of the endo-levanase E221A mutant. A schematic view of the protein in complex with levantetraose (magenta), colour is ramped from the N-terminus (blue) to C-terminus (red).

The bi-modular fold of the endo-levanase BT1760 (Fig. 1) is typical for GH32 family proteins^18–26^ to which the endo-levanase is allocated. The N-terminal domain of BT1760 harbouring the catalytic centre has a β-propeller topology of five β-sheets. The five propeller blades are comprised of antiparallel β-strands β1 to β19 and connected by loops of varied length. The C-terminal domain has a β-sandwich architecture. The N- and C-terminal domains are linked by loop 338–347 aa carrying an α-helix _339_PDAIDR_344_. The N- and C-termini of BT1760 are close to each other (Fig. 1).

### The N-terminal catalytic module: construction and verification of the catalytically inactive mutant

BT1760 was expressed in *E. coli* with its N-terminus truncated by a signal peptide of 21 aa: a codon for Ser22 in the original sequence (UniProt: Q8A6W6) was substituted with the ATG start codon as in^3,15^. The structure of crystallized wild-type protein was refined from Gly15, the first refined secondary structure element was the β1 strand accommodating a nucleophile Asp41. The N-terminal β-propeller catalytic module has a central cavity housing the active site with catalytic amino acids. As in most GH32 proteins^18^, the catalytic triad of BT1760 is composed of a nucleophile (Asp41), a transition state stabilizer (Asp169) and an acid/base catalyst (Glu221)^15,18^ creating an acidic environment for hydrolysis. Crystal structure of the wild-type BT1760 revealed a MES [2-(N-morpholino)ethanesulfonic acid] molecule in the catalytic centre with the morpholine ring of the ligand pointing towards the bottom of the active site. The morpholine ring was coordinated by two water molecules and the carbonyl oxygen of Thr104 while the ethanesulfonic acid ‘tail’ of MES was stabilized by side chains of Gln239 and Arg244. We assayed the possibility that MES may competitively inhibit levan degradation by BT1760, but no inhibition was observed (Supplementary Table S2).

In endo-levanase the Glu221 was assumed to donate a proton to the leaving fructosyl group during the hydrolysis reaction. So, in the E221A mutant, the substrate was expected to remain tightly bound to the enzyme. The E221A mutant was proved catalytically inactive: its catalytic activity towards levan (measured according to the reducing sugar release) was reduced by about 4,000 fold compared to the wild-type enzyme (Supplementary Table S3). Similar decline in activity was observed for the acid/base catalyst mutant of levansucrase in which the *k*_cat_ value was reduced 5,000 fold^27^. TLC analysis confirmed inactivity of the E221A – no FOS was produced from levan even after 24 h of incubation (Supplementary Fig. S1).

### Binding of levantetraose to the active site of the endo-levanase and hydrolysis of short levan oligomers

**Figure 2:**
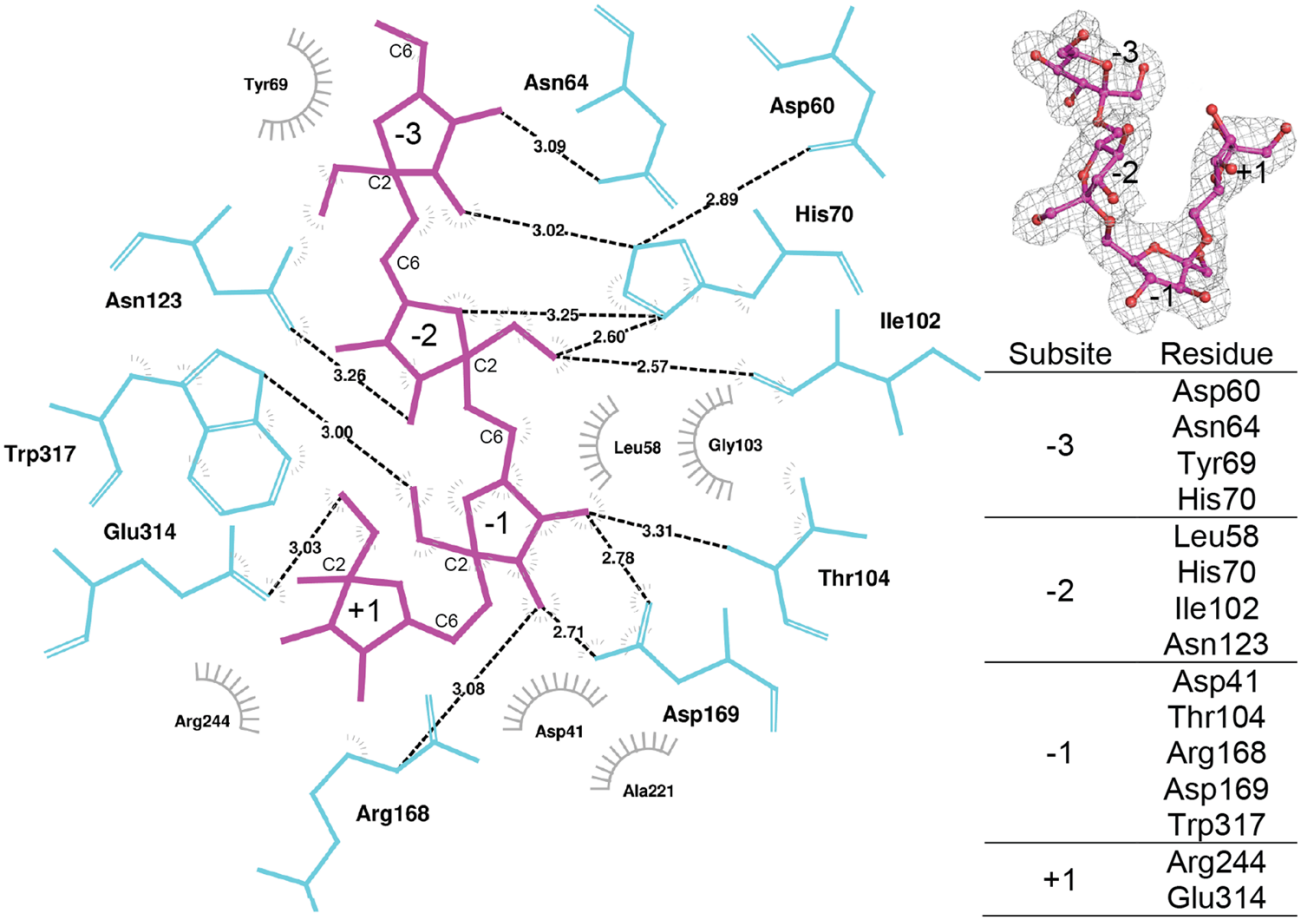
Binding of levantetraose to endo-levanase E221A variant. Amino acids in the vicinity of the ligand are in turquoise and the levantetraose bound at four subsites is in magenta. The 2*mF*_*o*_*-DF*_*c*_ electron density map of levantetraose contoured at 1σ is shown in grey (upper right). The residues of endo-levanase constituting the subsites of the ligand-binding pocket are listed on the right. LigPlot+^61^ was used for visualization of protein-ligand interactions.

Levantetraose with the degree of polymerisation of 4 (DP4) for soaking experiment was isolated from reaction products of BT1760 with timothy grass levan (see Supplementary Fig. S2). Unlike bacterial levans, timothy grass levan is unbranched^28^ that ensures the homogeneity of the levan oligomers. The ligand-bound structure of BT1760 revealed four subsites for the binding of fructose residues. According to sugar-binding subsite designation as recommended by^29^, three ‘minus’ subsites and one ‘plus’ subsite were specified in the active site. The nucleophile Asp41 is located just below the fructose residue bound at −1 subsite, ready to attack the anomeric carbon (C2) (Supplementary Fig. S3). The residues surrounding the active site comprise the binding subsites −2, −3 and +1 (Fig. 2). No rotameric changes but only minor side chain shifts (up to 0.5 Å) were detected in the binding pocket compared to the wild-type structure model, thus substrate binding likely does not induce any major conformational changes. An exception is the acid/base catalyst Glu221, which might convert into another rotamer to position itself optimally with respect to the glycosidic oxygen between the fructoses in −1 and +1 subsites. Since this carboxylic moiety is missing in the E221A mutant, the neighbouring Gln239 was found in two alternate conformations partially filling the artificial void (Supplementary Fig. S3). The electron density map is somewhat ambiguous in this part and does not allow the placement of any waters that might participate in the hydrolysis reaction (Supplementary Fig. S3).

The glycosidic bond is hydrolysed between the fructose residues bound at subsites −1 and +1. The distance between the nucleophile Asp41 and the general acid/base Glu221 in the structure of BT1760 is 5.2 Å suggesting double displacement mechanism of the endo-levanase reaction with retention of the configuration of the anomeric carbon atom. This mechanism is characteristic also for other GH32 family enzymes^18^. It should be noted that Thr104, Gln239 and Arg244 of BT1760 participated also in binding of MES: the morpholine ring of MES was bound at −1 subsite and the ethanesulfonic acid tail at +1.

We then analysed the hydrolysis of timothy grass levan oligomers of levantriose (DP3) to levanpentaose (DP5) by wild-type BT1760. TLC analysis of reaction products (Supplementary Fig. S2) showed that levantriose was the shortest levan oligomer cleaved by BT1760, levanbiose (DP2) was not degraded even after 22 hours of incubation. Initial events of L-FOS degradation were following: DP3 was degraded to fructose (DP1) and DP2; DP4 to DP1 and DP3; DP5 to mostly DP3 and DP2 (Supplementary Fig. S2). Later, DP3 resulting from DP4 and DP5 degradation was hydrolysed to DP1 and DP2. So, DP3 was formed as an initial product of both DP4 and DP5 hydrolysis. Considering levan degradation kinetics, DP3 was detected as a prominent product formed at the rapid phase of levan degradation whereas DP2 accumulated at the end of the reaction^15^. The accumulation of a DP3 fructan oligomer is described also in the case of inulin degradation by *A. ficuum* endo-inulinase^16^. Most probably, binding of levan oligomer at more than one ‘minus’ subsites is required for efficient catalysis thereby ruling out reaction with DP2.

### Comparison of the loop regions of endo-levanase BT1760 and endo-inulinase INU2

**Figure 3:**
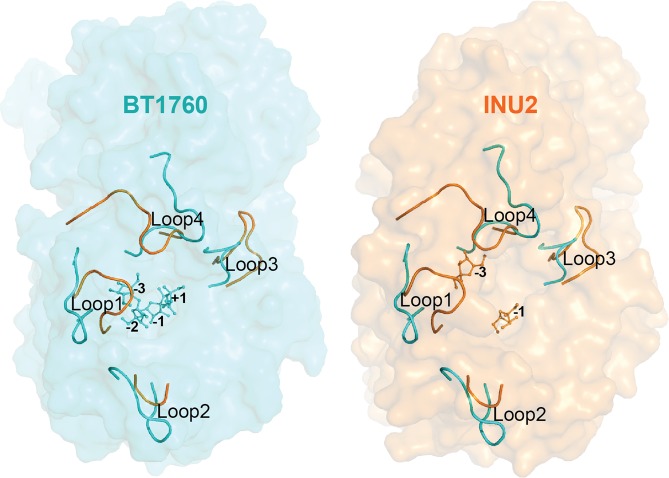
Structural alignment of endo-inulinase and endo-levanase. The surface view of endo-levanase BT1760 complexed with levantetraose (PDB: 6R3U) is shown in turquoise (left panel), the surface view of endo-inulinase INU2 complexed with two fructose residues (PDB: 3RWK) is shown in orange (right panel). Loops 1 (61–71 aa), 2 (124–133 aa), 3 (239–245 aa) and 4 (307–318 aa) of endo-levanase BT1760 and respective loops of endo-inulinase INU2 located at 62–70 aa, 130–133 aa, 257–267 aa and 320–330 aa are shown in both panels.

Following nomenclature of the loops of the β-propeller domain of the *A. ficuum* endo-inulinase INU2^16^, loop1 _61_YRPNPEATYHP_71_ of BT1760 is formed of 11 amino acids and loop2 _124_KFKPSSDQNA_133_ of 10 aa. Loop3 _239_QASFMRK_245_ of BT1760 is short and loop4 _307_NGNVGDVEPEWA_318_ is slightly longer (12 aa) (Fig. 3) and partially overlaps with the C-terminal β-sandwich module. Loop4 of BT1760 covers the edge of the C-terminal module, and the inner loop of 262–284 positions lines the interface of the two domains and contacts the C-terminal β-sandwich module. When comparing the structures of exo- and endo-acting inulinases, variability in loop regions was shown responsible for the width and shape of the substrate-binding pocket^16,21^. So, the loops 1 (62–69 aa) and 4 (317–326 aa) of INU2 were considered responsible for the wide opening of the substrate binding pocket^16^ enabling accommodation of a longer region of the inulin chain (Fig. 3).

**Figure 4:**
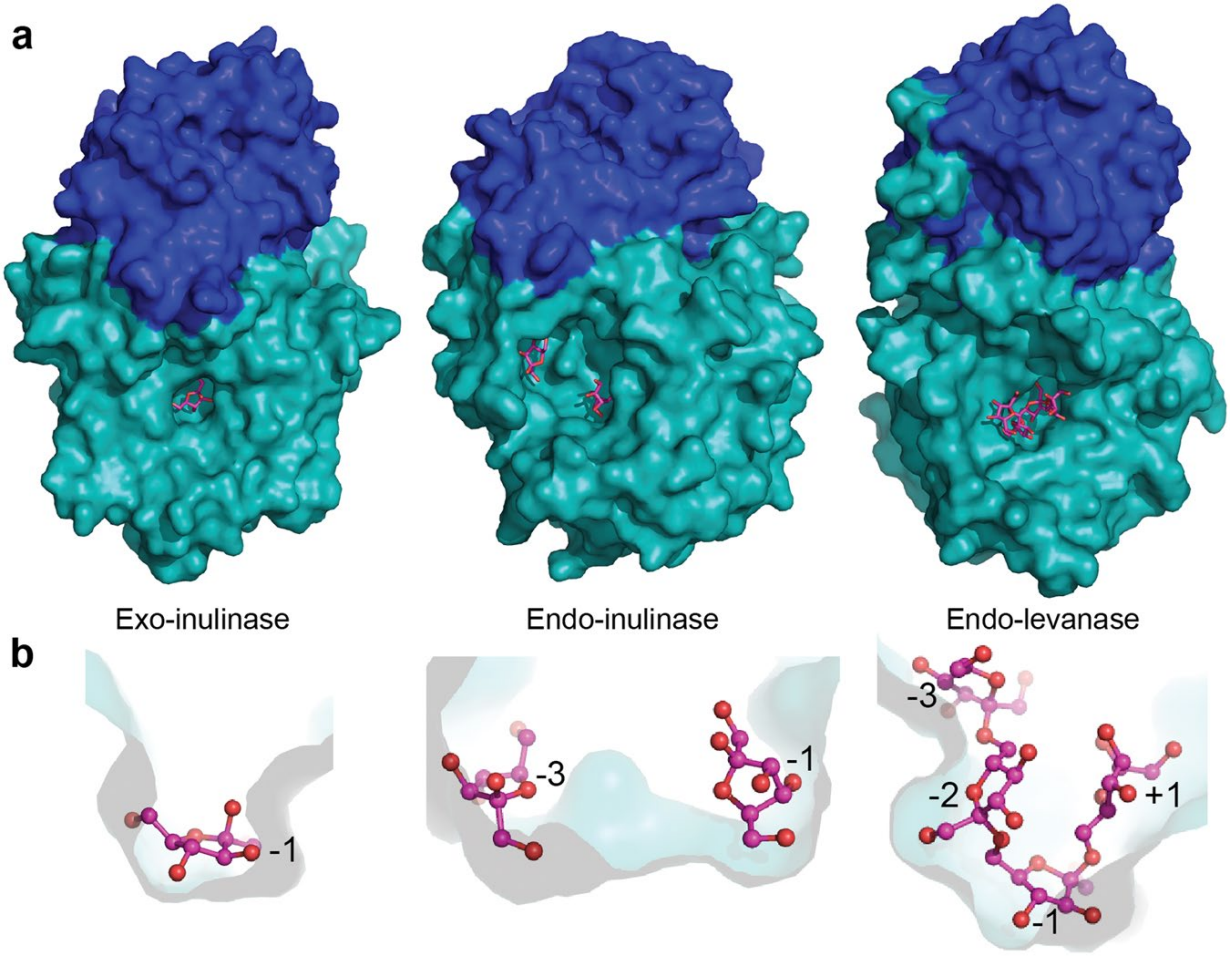
The ligand-bound structures of exo-inulinase, endo-inulinase and endo-levanase. The N-terminal catalytic β-propeller modules are shown in turquoise, the C-terminal β-sandwich domains in dark blue and the ligands in magenta. (**a**) 1Y9G, *Aspergillus awamori* exo-inulinase INUE with fructose in the active centre; 3RWK, endo-inulinase INU2 of *A. ficuum* with two fructose residues in the active centre and endo-levanase of *Bacteroides thetaiotaomicron* (6R3U) with levantetraose in the active centre. (**b**) Cross-sections of the substrate-binding pockets of respective fructanases, showing the positioning of ligand monomers. The binding subsites are marked with numbers.

The alignment of endo-levanase BT1760 and endo-inulinase INU2 structures revealed differences in the shape of the substrate binding pocket (Fig. 4, panel b). Compared with the loop regions of endo-inulinase, loops 1 (61–71 aa) and 4 (307–318 aa) of endo-levanase are pushed even further towards the sides of the β-propeller fold, while loop3 (239–245 aa) resides closer to the active site (Fig. 3). This arrangement gives the substrate-binding cavity of endo-levanase a shape of a bowl, while that of endo-inulinase reminds a flat-bottom washbasin with perpendicular edges (Fig. 4, panel b). The position of loop2 is quite similar in these two endo-acting enzymes (Fig. 3). Compared with exo-inulinase INUE, the active site cavities of the two endo-acting fructanases are wider and accommodate more than one fructose residue (Fig. 4)^21^.

As reported by^16^, Trp residues near loops 1 and 4 and within these loops may define the borders of the substrate-binding pocket. Active site structures of both endo- and exo-inulinases reveal closely positioned Trp residues. In exo-inulinase INUE, Trp38, Trp65 and Trp335 are pointed towards the active centre narrowing the substrate binding cavity at the bottom region, whereas in endo-inulinase INU2, positioning of tryptophans leaves space to accommodate more than one fructose residue at the bottom of the cavity^16,21^. The docking experiments^16^ confirmed that the active centre of INU2 may accommodate at least three fructose residues from kestopentaose (I-FOS, FFFFG). The fructose residues binding at subsites −3, −2 and −1 were positioned at the bottom of the active site pocket, while the fructose and glucose residues at +1 and +2 were shown lining the pocket edge. The −3 subsite was bordered by Trp40, leaving the enzyme with the ability to produce mainly inulotriose as a reaction product^16^.

**Figure 5:**
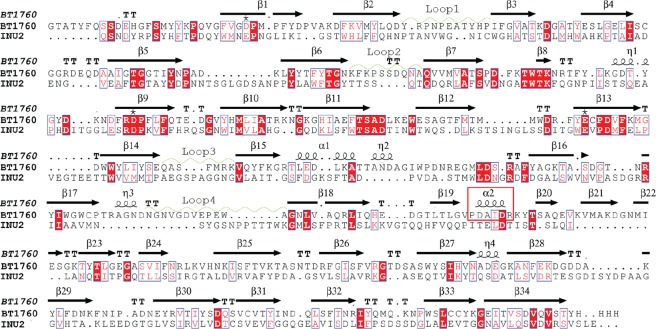
The sequence alignment of endo-levanase BT1760 (PDB: 6R3R) with endo-inulinase INU2 (PDB: 3RWK). The alignments are based on the superimposed models created in Dali server and visualized in ESPript Server http://espript.ibcp.fr^30,60^. Secondary structure elements of BT1760 are shown above the alignment. β-strands are shown by black arrows and helical structures by coils. Loops 1–4 of BT1760 corresponding to respective loops in INU2 structure^16^ are indicated by light green waves. Catalytic amino acids are designated by asterisks. The red frame shows the helix connecting N- and C-terminal domains.

Importantly, loop1 of BT1760 has no tryptophans (Fig. 5) and it reaches the side of the β-propeller fold. The levantetraose-bound structure of BT1760 shows that the −1 subsite accommodates a fructose residue that lies almost parallel to the bottom of the pocket defining the deepest-located (designated by us as level 0) binding subsite for a fructose residue (Fig. 4, panel b). The bottom of the substrate-binding pocket of BT1760 is formed by the side-chains of Asp41, Thr104 and Cys222. The +1 and −2 binding sites for fructose residues are positioned at the level above it (level 1) residing at the opposite slope of the cavity. The −3 subsite for fructose binding is located at level 2 close to the pocket opening. So, fructose residues of levantetraose were detected binding to the substrate pocket at three depths. Applying an analogous binding site description to INU2, the −3, −2 and −1 binding subsites of INU2 are all located at level 0 and the +1 subsite is uplifted to level 1. Differences in the architecture of substrate-binding pockets between INU2 and BT1760 most probably reflect the differences between the structures of L-FOS and I-FOS. The exo-acting enzymes such as exo-inulinase (see Fig. 4, panel b), possess a funnel-like active centre, which accommodates only one sugar monomer at level 0^16,21^.

### Structure of the C-terminal domain of BT1760

The C-terminal domain of BT1760 has a β-sandwich architecture of two facing β-sheets consisting of seven (β21, β24, β26, β27, β28, β29, β33; the upper sheet) and eight (β20, β22, β23, β25, β30, β31, β32; β34; the lower sheet) antiparallel β-strands (Figs 1 and 5). A concave surface is formed on the top sheet at the same face of the protein which harbours the active centre (Figs 1 and 4).

**Figure 6:**
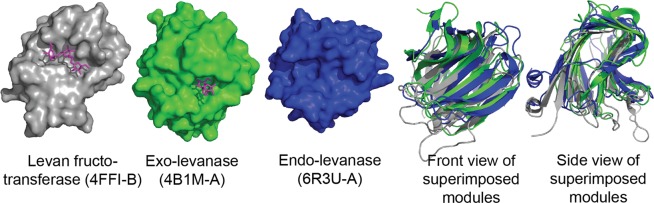
Comparison of C-terminal domains of three GH32 family fructanases. From left: the C-terminal β-sandwich module of levan fructotransferase of *Paenarthrobacter ureafaciens* (4FFI-B, 364–520 aa) with bound levantriose, the C-terminus (4B1M-A) of *Bacillus subtilis* exo-levanase with bound levanbiose, the C-terminal β-sandwich module of *Bacteroides thetaiotaomicron* endo-levanase (339–508 aa, PDB 6R3U-A). Front and side views of superimposed structures are shown on the right.

The Dali server^30^ search revealed BsCBM66 (PBD: 4AZZ) as the closest structural match to the β-sandwich domain of BT1760_339-506_ with a Z-score of 16.8 and sequence identity of 11% (Fig. 6, Supplementary Table S4). BsCBM66 is the C-terminal non-catalytic module of *B. subtilis* exo-levanase. BsCBM66 folds into two β-sheets containing six and seven antiparallel β-strands^17^. This module binds the non-reducing end of the levan chain providing the catalytic domain a high specificity for levan^17^. Supplementary Table S4 lists the data on five best structural matches to the C-terminal β-sandwich domain of BT1760. Aside of BsCBM66, the list includes two lectins: a mannose-specific lectin from *Homo sapiens* (PDB: 4YGB) and a carbohydrate-binding lectin from *Phaseolus vulgaris* (PDB: 1AVB), an α-amylase inhibitor from *P. vulgaris* (PDB: 1VIW) and a hypothetical protein BT3469 of *B. thetaiotaomicron* (PDB: 4JQT). In the case of BT3469, only 151 aa out of 431 occurred superimposable with the BT1760 structure.

From structure comparison data we conclude that the structure of C-terminal β-sandwich domain of *B. thetaiotaomicron* endo-levanase is novel to GH32 proteins and does not have close relatives among other structures in the PDB database. In contrast to CBM66 of *B. subtilis* exo-levanase and levan fructotransferase of *Paenarthrobacter ureafaciens*, no electron density was recorded for the ligand in the β-sandwich domain of BT1760.

### Sequence and structure comparison of BT1760 with other GH32 enzymes

According to the CAZy database, 13 structures of GH32 proteins are available. We extracted respective protein sequences from the databases and aligned with the Clustal Omega program. Sequence identity matrix of the proteins is presented in Supplementary Fig. S4. The sequence identity between BT1760 and other GH32 family proteins of the dataset occurred rather low, varying from 14.1 to 20.6%. The highest identity score was recorded against *B. subtilis* exo-levanase SacC (UniProt: P05656), the lowest against *Xanthophyllomyces dendrorhous* β-fructofuranosidase (UniProt: B8YJM2). The most conserved regions of aligned proteins were located around the catalytic triad: the nucleophile, the stabilizer from the RDP motif and the acid/base catalyst. For BT1760, respective amino acids are Asp41, Asp169 and Glu221 (see Fig. 5). For Glu221, the key function in catalysis was experimentally proven (see Supplementary Table S3 and Fig. S1). The nucleophile and general acid/base residues in sequences of these enzymes aligned well, confirming retaining mechanism for the catalysis as in other GH32 family proteins.

Expectedly, when the structure of the whole BT1760 molecule was compared against all protein structures in the Dali PDB90 database^30^, the GH32 enzymes were revealed as closest structural matches to BT1760. Invertase from *Thermotoga maritima* (PDB: 1UYP) had the highest Z-score (33.6), 394 aa Cα from 432 were superimposable, the identity between the sequences was 16% (Supplementary Table S4). Interestingly, the levan fructotransferase from *P. ureafaciens* (PDB: 4FFG) and the endo-inulinase from *A. ficuum* (PDB: 3RWK) had the same Z-score of 32.6 when compared to the BT1760 structure. Sequence identity was higher (18%) in the case of *P. ureafaciens* enzyme (Supplementary Table S4). In addition to above-mentioned enzymes, the top five list of structural matches included exo-inulinase of *A. awamori* (PDB: 1Y4W, Z-score 30.9) and invertase of *Arabidopsis thaliana* (PDB: 2AC1, Z-score 30.5).

### Separation and reassembly of N- and C-terminal modules of BT1760

#### Separation of the modules is detrimental to catalytic activity, stability and levan-binding ability of the protein

According to the Pfam database, BT1760 has a C-terminal β-sandwich domain of unknown function (DUF4975)^15,31^. Quite recently, a non-catalytic C-terminal β-sandwich domain of *B. subtilis* exo-levanase SacC was shown responsible for specific binding of levan and was defined as a founding member of the CBM family 66^17^. As the structure of the C-terminal domain of BT1760 reminded that of a CBM, we assayed the function of this domain. We dissected the N- and C-terminal domains of BT1760 and expressed as separate proteins. The crystal structure of BT1760 revealed an α-helix (_339_PDAIDR_344_) between the N- and C-terminal modules (Fig. 5), thereby we constructed two variants of these single-domain proteins (with and without this helix), expressed in *E. coli* and purified. The single-domain protein variants created in this study were designated as BT1760_1-349_, BT1760_1-338_, BT1760_340-508_ and BT1760_348-508_ (Supplementary Table S5). All four single-domain variants were unable to bind and hydrolyse levan. So, specific activity of the two N-terminal modules on 5 g/L *Pseudomonas syringae* levansucrase-produced levan was reduced by about 4,000 fold compared to the wild-type BT1760. Quite similar reduction of catalytic activity was recorded for the acid/base catalyst replacement mutant E221A (Supplementary Table S3).

#### Separated N- and C-terminal domains of BT1760 do not bind levan

The levan-binding ability of the proteins was assayed using two methods: (i) electrophoresis through native polyacrylamide gel containing 0.1% of levan and (ii) size-exclusion chromatography of proteins which were loaded onto the column with and without levan. Three different levans (Ps_S, Ps_R and Hs) described in Materials and Methods were used in the experiments. Dahlia inulin was used as a negative control since inulin is not a substrate for BT1760^15^. Proteins with catalytic activity were not used in this experiment as degradation of levan during the experiment may interfere with the assay.

Supplementary Fig. S5 (panel a) indicates strong levan binding ability for only two proteins: i) the catalytically inactive mutant E221A of BT1760 and ii) BsCBM66 – the levan-binding module of *B. subtilis* exo-levanase^17^ that was used as a positive control in the experiment. Electrophoretic mobility of BsCBM66 was strongly retarded by all levans, but only very slightly by inulin. Importantly, levan and inulin did not slow down electrophoretic mobility of single-domain variants of BT1760 – BT1760_1-349_ and BT1760_340-508_.

To evaluate the integrity of proteins in solution, a size-exclusion column (SEC) packed with Sephacryl S-200 was used (Supplementary Fig. S5, panel b). The upper panel of Supplementary Fig. S5 (b) shows that four proteins: BT1760, E221A mutant of BT1760, BsCBM66 and BsCBM66-BT1760 eluted from the column as a single peak. According to the SEC methodology^32^, proteins with higher molecular weights are eluting earlier, which is clearly seen for above-mentioned endo-levanase constructs having retention times of 44 min (BsCBM66-BT1760), 48 min (BT1760 and E221A mutant), and 54 min (BsCBM66). Calculated M_w_ values of the proteins are given in Supplementary Table S5. Variants of BT1760 comprising only N- or C-terminal domain were prone to aggregation and precipitation. So, the N-terminal domain variants BT1760_1-349_ and BT1760_1-338_ eluted from the column at 47 and 48 min that is earlier than expected from their M_w_. The C-terminal domain variants BT1760_340-508_ and BT1760_348–508_ eluted also earlier (at 49 and 48 min respectively) than calculated from their M_w_ and showed multiple elution peaks that may refer to protein aggregation. All three levans used in the experiment had some UV-absorbance at 280 nm and they eluted from the column with retention time around 30 min (Supplementary Fig. S5, panel b). When the BT1760 E221A mutant and BsCBM66 proteins and levans were co-loaded onto the SEC column, the complex eluting at 30 min was present at much higher intensity whereas no peak for the protein was observed showing that these proteins co-eluted with levan. From the affinity electrophoresis and the SEC analysis results (Supplementary Fig. S5) we conclude that E221A mutant and BsCBM66 bind levan very well whereas the separately expressed domains of BT1760 have no levan-binding ability.

#### The mixture of N- and C-terminal modules recovers slight activity

**Figure 7:**
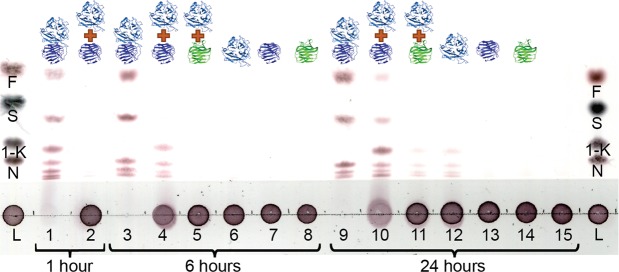
Co-incubation of N- and C-terminal modules restores slight endo-levanase activity. Proteins were incubated with 5 g/L Ps_S levan and samples withdrawn at indicated time were spotted onto a TLC plate as follows. Lanes 1, 3 and 9 – samples of reaction with BT1760; lanes 2, 4 and 10 – samples of reaction with the 1:1 mixture of BT1760_1-349_ and BT1760_348-508_; lanes 5 and 11 – respective samples of reaction with the 1:1 molecular ratio of BT1760_1-349_ and BsCBM66; lanes 6 and 12 – samples of reaction with the N-terminal BT1760_1-349_ module and lanes 7 and 13 with the C-terminal BT1760_348-508_. Lanes 8 and 14 shows samples from the reaction with BsCBM66. Lane 15 shows levan incubated during 24 hours under same conditions, but without the addition of enzymes or modules. The first and the last lane indicate the marker sugars: fructose (F), sucrose (S), 1-kestose (1-K), nystose (N) and levan (L). The scanned image is not digitally modified.

Surprisingly, co-incubation (in 1:1 molecular ratio) of N-and C-terminal modules of BT1760 in buffer with levan resulted in low but clearly recordable levan hydrolysis. Figure 7 shows that if BT1760_1-349_ and BT1760_348-508_ were co-incubated with 5 g/L of Ps_S levan, FOS were produced. In 6 hours, wild-type BT1760 had converted the majority of the substrate into FOS (Fig. 7, lane 3)^15^. At the same time point, only slight levan-degrading activity was observed in the BT1760_1-349_ and BT1760_348-508_ co-incubation experiment (Fig. 7, lane 4). After 24 h-incubation, however, both levan degradation and FOS production were clearly seen. Importantly, the catalytic module on its own (BT1760_1-349_) also produced FOS from levan by 24 hours of reaction (lane 12). Similar trace amount of FOS production was visible in the case of co-incubation of BT1760_1-349_ and BcCBM66 (1:1 molecular ratio; Fig. 7, lane 11). The C-terminal module of BT1760 as well as BsCBM66 did not produce FOS from levan during 24 h of incubation (Fig. 7, lanes 13-14).

#### Addition of BsCBM66 to the N-terminus of BT1760 has no effect on catalytic activity of BT1760

The wild-type endo-levanase has a K_m_ of 13.6 g/L towards Ps_S levan, and a k_cat_ value of 466.8 1/s^15^. BT1760 is the most potent endo-levanase described so far, cleaving levan 300 times faster than the endo-levanase from *Bacillus licheniformis*^15^. We asked, can endo-levanase BT1760 be further improved by adding a levan-binding CBM to it? Up to now, only one non-catalytic levan-binding module has been characterized – the CBM66 of *B. subtilis* exo-levanase SacC. This module has a high affinity for L-FOS and levan and it accommodates an oligosaccharide of at least two fructose residues in its binding site^17^. However, when BsCBM66 was N-terminally added to full-length BT1760, the activity of the CBM-amended endo-levanase was similar to that of the wild-type BT1760. According to catalytic efficiency, BT1760 slightly prefers Hs levan (42.7 1/s × g/L), following Ps_S (34.3 1/s × g/L) and Ps_R levans (30.1 1/s × g/L) and BsCBM66-BT1760 has the same order of levan preference (50.6, 35.8, 30.4 1/s × g/L respectively) (Supplementary Table S6). We conclude that the N-terminally added BsCBM66 neither enhances nor hinders levan degradation by BT1760.

## Discussion

According to the CAZy database^18^, only 13 enzymes (14 if the BT1760 structure is included) from the GH32 family have resolved structures. This number is rather low if compared to some other families, for example GH13 (120 structures), GH5 (78 structures) and GH1 (63 structures). Aside from invertases and various fructosyl transferases, the GH32 family also includes exo- and endo-acting inulinases and levanases. The 3D structures are available for the exo-inulinase of *Aspergillus awamori*^21^, the endo-inulinase INU2 of *A. ficuum*^16^ and the β-sandwich module of the *Bacillus subtilis* exo-levanase^17^. The present paper describes the first crystal structure of an endo-levanase from *Bacteroides thetaiotaomicron* (EC 3.2.1.65). The enzyme has a bi-modular fold common to GH32 family proteins composed of an N-terminal five-bladed β-propeller and a C-terminal β-sandwich domain (Figs 1 and 4).

The levantetraose-bound structure of the E221A mutant of BT1760 suggests that the levan chain should be bent into the active site cavity to enable endo-cleavage. The fructose residue of levantetraose bound at the −1 subsite lies at the bottom of the cavity while the fructose residues contacting the −2 and +1 subsites are bound above the −1 subsite – closer to the active site opening (Fig. 4). Quite different allocation of the fructan ligand has been recorded for endo-inulinase: three fructose monomers of the ligand occupying the ‘minus’ subsites lie along the bottom of the cavity, while the two ‘plus’ subsites reach upwards^16^. Our data confirm that the loops lining and forming the edges of the active centre emanate structural differences between the substrate-binding cavities of exo- and endo-acting fructanases (Figs 3 and 4). We reckon that the fine-tuning of composition of surface loops ensures the linkage-specificity of an endo-fructanase – the ability to cleave either β-2,6 (as in levan) or β-2,1 (as in inulin) linkages.

Bacterial levans have typically a very high molecular weight (up to several megadaltons) due to their high DP whereas levans of plants have much lower molecular weight and DP^15^. For example, the levan from timothy grass that is an excellent substrate for BT1760^15^ has an average DP of 260^33^. The ligand-bound structure of endo-levanase revealed details of levantetraose (DP4) binding. However, if a high-molecular weight levan is hydrolysed, the first endo-cuts should be made into high-DP levan chains. We suggest that for initial endo-cuts, bending of the levan chain into the substrate-binding pocket is required. These initial endo-cuts ‘chop’ levan into oligomers of moderate DP. When these moderate-length levan oligomers bind with their non-reducing ends at −3 subsite, levantriose is produced. This model is in agreement with our earlier data showing that levantriose is a prominent product formed at the rapid phase of levan degradation by BT1760^15^.

Our results allow to conclude that the C-terminal β-sandwich domain of *B. thetaiotaomicron* endo-levanase is not a carbohydrate binding module. As the strongest proof for that we detected no levantetraose bound to this module in the crystallized protein – the only ligand molecule was found bound to the active site pocket of the catalytically inactive mutant of BT1760. Considering the GH32 enzymes with resolved structures, function of the C-terminal β-sandwich domain has mostly not been addressed. In the case of the catalytically inactive invertase of *T. maritima*, a bound ligand (raffinose) was detected only in the active site of the β-propeller fold^34^. The authors hypothesized that the C-terminal β-sandwich domain of the invertase has a role in stabilization of the protein. In kestopentaose-soaked crystals of endo-inulinase INU2, two fructose residues were detected bound in the active site, and no function was predicted for the C-terminal β-sandwich domain^16^. However, in the case of the catalytically inactive mutant of the *P. ureafaciens* fructosyl transferase, levanbiose was identified bound in the active site pocket of the β-propeller fold as well as on the concave surface of the C-terminal β-sandwich domain (Fig. 6)^24^. The authors proposed that while levan chain binds to the C-terminal domain of the protein, its nonreducing end reaches into the active site for catalysis.

We suggest that the C-terminal β-sandwich domain of endo-levanase BT1760 is required for correct folding, stability and solubility of the protein. Shen *et al*. (2015) have shown that the C-terminal domain (composed of two antiparallel β-sheets) of an α-glucosidase stabilizes the catalytic domain through hydrophobic contacts between the surface areas of the two domains^35^. By screening the surface of the BT1760 N- and C-terminal domains, we detected hydrophobic patches at the interface of the two domains that may indeed contribute to tight packing and stabilization of the protein. We also showed that if the two modules were separately expressed, they tended to aggregate which can be due to exposed hydrophobic surface. However, when separately expressed N- and C-terminal modules were co-incubated with levan, a low but clearly detectable levan-degrading activity emerged (Fig. 7). We hypothesize that the two modules may bind with each other through hydrophobic surface contacts yielding a structure with levan-degrading ability.

**Figure 8:**
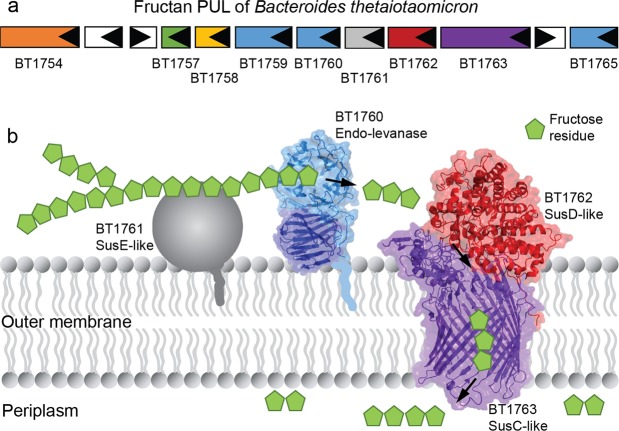
A model of initial events of levan utilization by *B. thetaiotaomicron*. (**a**) Genomic arrangement of levan utilization locus (the fructan PUL). (**b**) A proposed schematic model of levan binding, hydrolysis and transport of hydrolysis products into the periplasm. The SusE-like membrane anchored BT1761^3^ is suggested to mediate levan binding, while the endo-levanase (BT1760) cleaves the fructan polymer into fructo-oligosaccharides (L-FOS). The L-FOS are transported into the periplasm via SusC- and SusD-like complex of BT1762 and BT1763 proteins (PDB: 5T3R;^36^).

BT1760 is bound to the outer surface of *B. thetaiotaomicron*^3^. So, levan-degrading *B. thetaiotaomicron* can supply L-FOS for other gut community members if levan is present in the diet. Since endo-levanase itself has no levan-binding module, it may require ‘help’ from other neighbour proteins binding levan at the surface of the bacterium (Fig. 8). Indeed, the fructan PUL of *B. thetaiotaomicron* encodes two non-catalytic outer membrane anchored proteins: BT1761 (a SusE homologue) and BT1762 (a SusD homologue) that specifically bind levan^3,36^. Genomic disruption of BT1762 in *B. thetaiotaomicron* strongly reduced the growth of the bacterium on levan, but did not affect extracellular hydrolysis of levan^3^. The crystal structure of SusCD-like complex of *B. thetaiotaomicron* revealed a ‘pedal bin’ mechanism for transport of the substrate by the complex. According to it, the empty outer membrane transporter (BT1763, SusC-like) is covered with a mobile SusD-like lid sampling a range of conformational states^36^. When the substrate (ligand) binds, the lid covers the transporter and entraps the ligand. Following TonB-dependent events result in ligand transport into the periplasm^36^. We hypothesize that BT1761 is required to support binding and proper accommodation of levan chain in the substrate-binding pocket of the endo-levanase. The released L-FOS are captured by the BT1762 (lid) and further transported into the periplasm (Fig. 8).

## Materials and Methods

### Gene cloning and protein expression. Construction of endo-levanase variants

The native (wild-type) BT1760 is 523 aa long. We cloned and expressed wild-type BT1760 without the N-terminal signal peptide and added a His_x6_-tag to the C-terminus as described in^15^. The length of the expressed wild-type BT1760 is 508 aa. All endo-levanase variants constructed in this work were derived from this BT1760 protein and expressed with the C-terminal His_x6_-tag. E221A mutation was introduced into the wild-type BT1760 protein by site-specific mutation^27^ of the *BT1760* gene (see Supplementary Table S5 for mutagenic oligonucleotides). The *BsCBM66* gene was amplified from genomic DNA of *B. subtilis* 168 (DSM 23778; DSMZ, Germany) with primers presented in Supplementary Table S5. Genetic manipulations used in this work are described in Supplementary Table S5 in more detail.

*E. coli* BL21(DE3)^37^ was used as the host for the expression. A simplified autoinduction medium^38^ was used for protein overproduction: the LB-based medium was supplemented with 25 mM phosphate buffer (Na_2_HPO_4_/KH_2_PO_4_; pH 7.2) and 0.3% *v/v* glycerol to which sugars 0.025% w*/v* glucose and 0.1% w*/v* lactose were added. Ampicillin (150 µg/mL) or kanamycin (100 µg/mL) were supplemented for plasmid preservation. Cells were first grown overnight in LB medium and then diluted 100 times to autoinduction medium in which they were first grown during 2 hours at 37 °C following by overnight incubation at 22 °C. For protein crystallization, the cells were grown in 1 L of the autoinduction medium, for the purification of endo-levanase mutants, the cells were grown in 200 mL. Cells were harvested with centrifugation, washed and stored at −20 °C if not stated otherwise.

### Fructans used in the assays

Three different high-molecular weight levans described in^15^ were used in this study as substrates for the endo-levanase: 1) high-molecular levan synthesized from sucrose (Ps_S) by levansucrase Lsc3 of *Pseudomonas syringae* pv. tomato^15^, 2) high-molecular levan synthesized from raffinose (Ps_R) by levansucrase Lsc3^15^, 3) levan produced by *Halomonas smyrnensis* AAD6T (Hs)^39^. Dahlia inulin (Merck KGaA, Germany) was used as a negative control in fructan binding assays^15^.

To obtain the L-FOS of desired DP, levan from timothy grass^40^ kindly provided by Dr Anna Kasperowicz (Poland) was degraded with endo-levanase as in^15^. After 15 minutes of incubation, reaction was stopped by heating and the mixture of reaction products and residual substrate was separated by gel permeation chromatography: XK16/100 column filled with BioGel P2 (more details in Supplementary Fig. S2). The fractions of L-FOS with a DP 3, 4 or 5 were collected, dried and stored for further analysis at −20 °C. The obtained L-FOS were used as substrate in enzymatic assays and for soaking of crystals of BT1760 E221A mutant.

### Protein purification and enzyme activity assay

For crystallization trials, the pelleted *E. coli* cells overexpressing wild-type or E221A mutant variant of BT1760 were resuspended in IMAC buffer A (50 mM Na-phosphate, pH 6.0; 300 mM NaCl; 10 mM imidazole, pH 6.0) with cOmplete™, EDTA-free Protease Inhibitor Cocktail (Roche). Cells were disrupted with ultrasonication and centrifuged at 32 579 × *g* at 4 °C during 40 min. The resulting supernatant was filtered and loaded onto IMAC HisTrap^TM^ HP column. ÄKTAprime plus (GE Healthcare) system was calibrated with IMAC buffer A and the protein was eluted with an imidazole gradient from 10 to 600 mM. Samples containing desired protein were collected and concentrated up to 5 mL using Amicon Ultra-15 Centrifugal Filter (Merck). Size-exclusion chromatography (SEC) column Superdex® 200 (GE Healthcare) was equilibrated with SEC buffer (20 mM MES, pH 6.5; 150 mM NaCl) and the protein sample was applied to the column. After SEC the protein was concentrated to ~30 mg/mL and stored at −80 °C. All other proteins used in this work were purified after growing cells in 200 mL media using only IMAC purification. BT1760, E221A mutant, BT1760_1-349_, BT1760_1-338_ and BsCBM66-BT1760 were dialysed against McIlvaine’s buffer (pH 6.0) with 0.02% Na-azide^41^ due to their acidic pI (see Supplementary Table S5), and BT1760_340-508_, BT1760_348-508_ or BsCBM66 proteins against the CBM buffer (50 mM TRIS, pH 7.5; 300 mM NaCl; 0.02% w*/v* Na-azide).

Catalytic activity of endo-levanase and its mutants on levans was measured by recording the reducing sugar release in McIllvaine’s buffer (pH 6.0) at 37 °C. Kinetic parameters for BT1760 and BsCBM66-BT1760 were calculated from initial velocities of the reaction conducted at varied concentrations of the substrate. For details see^15^. At least two independent experiments with at least two technical replicates were conducted.

Thin layer chromatography (TLC) was used to visualize the pattern of products formed in endo-levanase reaction with levan^15^ or L-FOS (for details, see Supplementary Fig. S2). Reactions with different endo-levanase modules were conducted in 50 mM Na-phosphate buffer (pH 7.0) with 150 mM NaCl at 37 °C. In experiments presented in Supplementary Figs S1 and S2, McIllvaine’s buffer (pH 6.0) and reaction temperature of 37 °C was used. At fixed time points the samples were withdrawn and heated for 5 min at 96 °C to stop the reaction. Samples (0.5 µL) were spotted onto silica gel plates with concentrating zone (Millipore) and run twice with a solvent system of chloroform: acetic acid: water (60:70:10; *v/v/v*)^15^. Sugar spots were visualized by immersion of the plates in aniline–diphenylamine reagent and subsequent heating of the dried plates at 120 °C^42,43^.

### Protein crystallization

Wild-type and E221A mutant variant of BT1760 were crystallized using the vapor-diffusion method. Crystals were grown in hanging drops of 32 or 16 mg/mL protein sample (wild-type or E221A, respectively) and crystallization reagent in 2:1 ratio. The reagent for wild-type enzyme contained 16–22% (*w/v*) PEG 6000, 1 mM ZnCl_2_, 0.1 M MES-NaOH, pH 6.5, and for E221A mutant, 12–14% (*w/v*) PEG 6000, 0.5 mM ZnCl_2_, 0.1 M MES-NaOH, pH 6.5. Clusters of needles appeared overnight and matured in 3–4 days at various temperatures (4 °C or 9 °C or room temperature). Single crystals were dislodged from a cluster, dipped briefly into a drop of reservoir solution supplemented with 20% (*v/v*) glycerol for cryoprotection, and flash frozen in liquid nitrogen or placed directly into a 100 K cryostream. Crystals of E221A mutant were soaked overnight in reservoir solution supplemented with 4 mM levantetraose prior to cryoprotection and freezing.

### Data collection and structure determination

Highly redundant diffraction data was collected on a Rigaku Compact HomeLab diffractometer with a MicroMax-003 sealed-tube Cu-anode source (1.54-Å radiation), a 4-circle partial-chi goniometer, and a Saturn 944 + CCD detector. The data of altogether 1980 degrees were collected from a single crystal at 100 K and processed with XDS^44^ to 2.0 Å. The structure was solved by coupling molecular replacement and single-wavelength anomalous dispersion methods (MR-SAD) using the PHENIX software suite^45^. Firstly, MR was performed with Phaser^46^ and the MRage pipeline using an HHpred^47^ sequence alignment based on the sequence of BT1760 as input. The best ambiguous solution was obtained in space group I222 with endo-inulinase from *Aspergillus ficuum* (PDB: 3RWK) serving as the search model. This result was input to AutoSol^48^ as a partial model for experimental phasing with 20 anomalous scatterers (S atoms, alternatively Zn or Cl) specified. The initial solution was further improved by automatic building with AutoBuild^49^. Iterative refinement with phenix.refine^50^ and manual building with Coot^51^ yielded a working model with 493 of 508 residues placed (13 missing from N-, and 2 from C-terminus) and *R*_*work*_/*R*_*free*_ factors of 0.15/0.19.

A native dataset of wild-type BT1760 to 1.65-Å resolution was collected on beamline F1 at the Cornell High Energy Synchrotron Source (Ithaca, NY, USA) using a ADSC Quantum 270 detector. Diffraction data of the E221A mutant crystal soaked with levantetraose was collected on BL13-XALOC beamline^52^ at synchrotron ALBA (Barcelona, Spain) to a resolution of 1.90 Å on a Dectris Pilatus 6M detector. The data were processed with XDS. The previously obtained working model of the wild-type enzyme was fitted against the higher-resolution dataset as a rigid body using PHENIX. For the E221A mutant dataset, a refined wild-type model was used. Subsequent refinement and automatic solvent, ion and ligand placement were also performed in PHENIX, and manual building was done in Coot. Geometry restraints for levantetraose were generated with eLBOW^53^. TLS groups used in the later stages of refinement were calculated using the TLSMD web server^54,55^. Structure validation was performed with MolProbity^56^. Molecular graphics were prepared with PyMOL^57^. The atomic coordinates and structure factors of wild-type endo-levanase and E221A mutant have been deposited in the Protein Data Bank with accession codes 6R3R and 6R3U, respectively.

### Co-incubation of N- and C-terminal modules of BT1760 with levan

The N- and C-terminal modules (BT1760_1-349_ and BT1760_348-508_) of endo-levanase BT1760 were freshly purified before the assay. The purified modules were used/mixed in desired quantities. The concentration of 1 µM was used for BT1760, BT1760_1-349_, BT1760_348-508_, BsCBM66 and 1:1 µM concentration was used for BT1760_1-349_:BT1760_348-508_ and BT1760_1-349_:BsCBM66 combinations. The proteins were incubated in 50 mM Na-phosphate buffer (pH 7.0) containing 150 mM NaCl with 5 g/L of *P. syringae* levan (Ps_S), and at certain time points samples were withdrawn for TLC analysis. Experiments were repeated three times and a representative chromatogram is presented (Fig. 7).

### *In silico* methods

ExPASy Proteomics Server (http://expasy.org) was used to calculate the theoretical molecular weight extinction coefficient at 280 nm of C-terminally His_x6_-tagged endo-levanase and its constructs for protein concentration determination.

Protein sequences were obtained from UniProt database^58^ and aligned using the Clustal Omega tool^59^. Protein sequence identity matrix was retrieved from the alignment.

The protein structure alignment was conducted in the Dali Server against PDB90 database^30^. The structures of the BT1760 wild-type enzyme (PDB: 6R3R) and of the C-terminal β-sandwich domain BT1760_339-506_ were used as the bait. The alignment was visualized with the ESPript program http://espript.ibcp.fr^60^.

## Supplementary information

Supplementary Information

## Data Availability

All data generated or analysed during this study are included in this published article (and its Supplementary Information files) and deposited to PDB.
