# Author Correction: First crystal structure of an endo-levanase – the BT1760 from a human gut commensal *Bacteroides thetaiotaomicron*

**DOI:** 10.1038/s41598-019-54274-z

**Published:** 2019-11-20

**Authors:** Karin Ernits, Priit Eek, Tiit Lukk, Triinu Visnapuu, Tiina Alamäe

**Affiliations:** 1 Department of Genetics, Institute of Molecular and Cell Biology, University of Tartu, Riia 23, 51010 Tartu, Estonia; 2 Department of Chemistry and Biotechnology, Tallinn University of Technology, Akadeemia tee 15, 12618 Tallinn, Estonia

Correction to: *Scientific Reports* 10.1038/s41598-019-44785-0, published online 11 June 2019

The Supplementary Information file that accompanies this article contains errors.

In the legend of Supplementary Figure S3 where:

“The structure of wild-type endo-levanase (dark blue) is superimposed on the structure of E221A mutant (light blue). Levantetraose (magenta) and water molecules are from the E221A structure. Fructose-binding subsites, catalytic amino acids, two alternate conformations of Gln239, and C2, O6, and C6 from β-2,6-linkage are designated.”

should read:

“The structure of wild-type endo-levanase (light blue) is superimposed on the structure of E221A mutant (dark blue). Levantetraose (magenta) and water molecules are from the E221A structure. Fructose-binding subsites, catalytic amino acids, two alternate conformations of Gln239, as well as C2, O6 and C6 from β-2,6-linkage are designated.”

In the legend of Supplementary Figure S5 where:

“The images were not digitally modified except for applying grayscale mode, the originals are presented in Supplementary Figure S5.”

should read:

“The images were not digitally modified except for applying grayscale mode, the originals are presented in Supplementary Figure S6.”

