# Supplementary material for: First crystal structure of an endo-levanase – the BT1760 from a human gut commensal Bacteroides thetaiotaomicron
Source: Sci Rep. 2019 Jun 11;9:8443. doi: 10.1038/s41598-019-44785-0 (PMC6560043; doi:10.1038/s41598-019-44785-0)
Supplement: Supplementary file 1 — Supplementary Information [file 41598_2019_44785_MOESM1_ESM.pdf]

First crystal structure of an endo-levanase – the BT1760 from a human gut commensal *Bacteroides thetaiotaomicron*

Authors: Karin Ernits[1], Priit Eek[2], Tiit Lukk[2], Triinu Visnapuu[1] and Tiina Alamäe[1,*]

[1]Department of Genetics, Institute of Molecular and Cell Biology, University of Tartu, Riia 23, 51010 Tartu, Estonia

[2]Department of Chemistry & Biotechnology, Tallinn University of Technology, Akadeemia tee 15, 12618 Tallinn, Estonia

*Corresponding author

E-mail: talamae@ebc.ee (TA)

Table S1. Data collection and refinement statistics

PDB code	BT1760 WT (MR-SAD phasing)	BT1760 WT 6R3R	BT1760 E221A + FRU_4 6R3U
**Data collection**			
Space group	I222	I222	I222
Cell dimensions			
a, b, c (Å)	77.40 112.26 172.50	77.66 110.61 171.24	78.11 112.33 174.14
α, β, γ (°)	90 90 90	90 90 90	90 90 90
Resolution (Å)	38.71-2.00 (2.07-2.00)*	29.79-1.65 (1.71-1.65)*	47.21-1.90 (1.97-1.90)*
R_{merge}	0.136 (0.826)	0.059 (0.764)	0.084 (1.129)
$I/\sigma I$	42.2 (6.6)	16.9 (2.3)	15.6 (1.9)
Completeness (%)	100.0 (100.0)	99.2 (99.3)	99.8 (99.2)
Redundancy	69.4 (41.1)	4.9 (4.9)	7.8 (7.9)
**Refinement**			
Resolution (Å)		29.79-1.65	47.21-1.90
No. reflections		87995	60564
R_{work}/R_{free}		0.154 / 0.171	0.161 / 0.190
No. non-hydrogen atoms		4500	4409
Protein		3971	4018
Ligand/ion		69	65
Water		460	326
B-factors		27.0	42.6
Protein		25.6	42.1
Ligand/ion		47.3	60.7
Water		35.9	45.8
R.m.s deviations			
Bond lengths (Å)		0.004	0.012
Bond angles (°)		0.77	1.03

*Statistics for the highest-resolution shell is shown in parentheses

Table S2. Inhibition of endo-levanase BT1760 by 2-(N-Morpholino)ethanesulfonic acid, MES

MES (mM)	Specific activity (%)
0	100.0 ± 4.0
10	95.5 ± 5.1
20	94.7 ± 6.3
30	96.8 ± 5.4
40	99.0 ± 4.5
50	97.6 ± 1.3
100	98.7 ± 1.0
200	100.4 ± 1.3

Specific activity of wild-type endo-levanase (BT1760) on 5 g/L Ps_S levan was measured in McIlvaine's buffer (pH 6.0) at 37˚C supplemented with different concentrations of MES. The activity of BT1760 (123.05 U/mg) recorded in the absence of MES was taken for 100%. Mean values ± standard deviation of two independent measurements are presented.

Table S3. Specific activities (U/mg) of endo-levanase constructs on 5 g/L Ps_S levan

Endo-levanase construct	U/mg	Activity reduced (times)
BT1760 (wild-type)	123.05±2.05	1
BT1760E221A	0.03±0.00	3973
$BT1760_{1-349}$	0.03±0.00	3633
$BT1760_{348-508}$	0.03±0.02	4258

Specific activity was measured as shown in Table S2, but no MES was added. Mean values ± standard deviation of at least two independent measurements are presented.

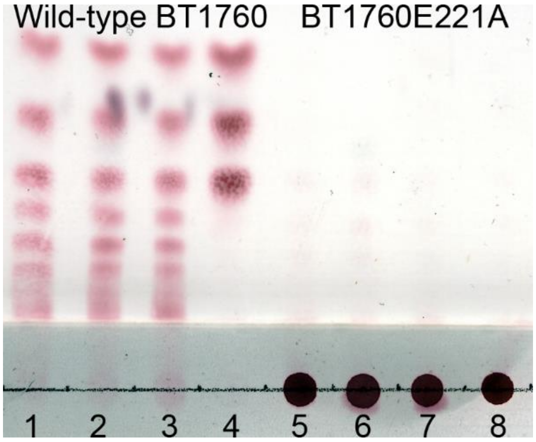

Figure S1. The E221A mutant of BT1760 loses the ability to degrade levan. Reaction was conducted in McIlvaine's buffer (pH 6.0) with 0.02% Na-azide at 37°C. 4.6 µg/mL of protein was incubated with 5g/L levans of different origin (Ps_S, Ps_R, Hs and timothy grass; for designation, see Materials and Methods) for 24 hours. TLC analysis was conducted to visualize the reaction products. Lanes 1-4, reaction of BT1760 with Ps_S, Ps_R, Hs and timothy grass levans, respectively; lanes 5-8, reaction of BT1760E221A with levans spotted in the same order. The scanned image is not digitally modified.

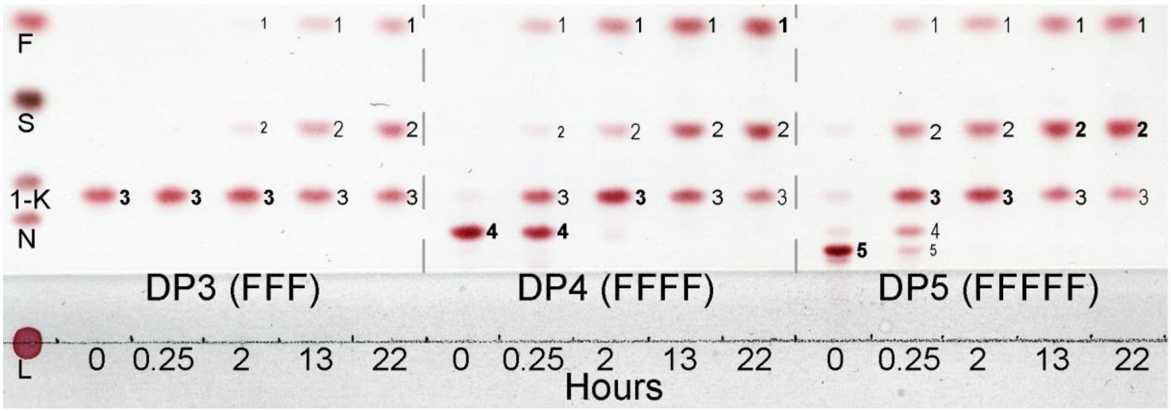

Figure S2. Hydrolysis of levantriose (DP3), -tetraose (DP4) and -pentaose (DP5) by endo-levanase BT1760. The mixture of 30 mM fructose (F), 30 mM sucrose (S), 8 mM 1-kestose (1-K), 8 mM nystose (N) and 7 g/L levan (L) was used as a marker. The numbers 1-5 indicate degree of polymerization (DP) of the fructan. Reactions of endo-levanase BT1760 (5 µg/mL) with L-FOS of DP3 (10 mM), DP4 (8 mM) and DP5 (6 mM) were conducted in McIlvaine's buffer (pH 6.0) with 0.02% Na-azide at 37°C. At time points shown in figure, samples were withdrawn and analysed using TLC. The scanned image is not digitally modified.

L-FOS used as a substrate for BT1760 were produced from 5 g/L timothy grass levan in reaction with BT1760 as in [1]. Protein-free samples were separated by gel permeation chromatography on BioGel P2 (BioRad) column (XK16/100, GE Healthcare; 16x900 mm) connected to ÄKTA prime plus chromatography system (GE Healthcare). Sample injection volume was 0.5 mL with the initial saccharide concentration of approximately 20 mg/mL. Flow rate was 0.1 mL/min with MQ water and 2 mL fractions were collected separately. The fractions containing reducing sugars were analyzed using TLC. Fractions containing levan oligomers of DP 3, 4 and 5 were collected, separately pooled and dried at 40°C using SpeedVac concentrator.

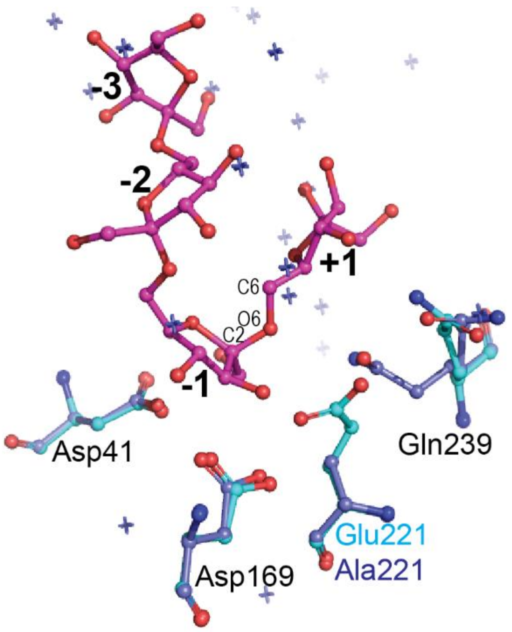

Figure S3. The alignment of apo- and ligand-bound structures of endo-levanase BT1760. The structure of wild-type endo-levanase (~~dark~~ blue) is superimposed on the structure of E221A mutant (~~light~~ blue). Levantetraose (magenta) and water molecules are from the E221A structure. Fructose-binding subsites, catalytic amino acids, two alternate conformations of Gln239, ~~and~~ C2, O6~~,~~ and C6 from β-2,6-linkage are designated.

Table S4. Protein structure comparison results from Dali server. The $BT1760_{15\text{-}506}$ or $BT1760_{339\text{-}506}$ structures were used as a query. Top 5 closest structures from the PDB90[2] database are shown.

PDB ID/Chain	Z-score	Seq. id (%)	Rmsd (Å)/Superimposed aa	Protein/organism
**Query $BT1760_{15\text{-}506}$**				
1UYP-A	33.6	16	2.7/394 from 432	Invertase from *Thermotoga maritima*
4FFG-A	32.6	18	2.5/405 from 480	Levan fructotransferase from *Paenarthrobacter ureafaciens*
3RWK-X	32.6	15	2.9/422 from 493	Endo-inulinase from *Aspergillus ficuum*
1Y4W-A	30.9	15	2.8/424 from 517	Exo-inulinase from *Aspergillus awamori*
2AC1-A	30.5	17	2.7/427 from 537	Cell-wall invertase from *Arabidopsis thaliana*
**Query $BT1760_{339\text{-}506}$**				
4AZZ-A	16.8	11	2.5/148 from 165	Carbohydrate binding module CBM66 from *Bacillus subtilis*
4YGB-A	14.7	13	2.6/148 from 192	Mannose-specific lectin from *Homo sapiens*
1AVB-A	14.6	9	2.9/150 from 226	Carbohydrate-binding lectin from *Phaseolus vulgaris*
4JQT-A	14.5	9	2.6/151 from 431	Putative glycosyl hydrolase (BT3469) from *Bacteroides thetaiotaomicron*
1VIW-B	14.4	7	3/146 from 198	Alpha-amylase inhibitor from *Phaseolus vulgaris*

	B8YJM2_Xd_FF	F1ADK9_Aj_FT	G7XM46_Ak_FTF	Q8A6W6_Bt_Bt1760	E3PQS3_Pt_S6FTF	Q43866_At_INV1	Q93X60_Ci_FF	A2TLS9_Bl_FF	O33833_Tm_Inv	Q9KJD0_Pu_FTF	W8GV60_Bl_LevB1	P00724_Sc_INV2	E5D0X5_So_INV	O94220_Af_INU2	Q96TU3_Aa_INUE	P05656_Bs_SACC
B8YJM2_Xd_FF	100	35.2	34.6	14.1	20.8	23.6	20.7	19.7	24.4	17.0	16.7	18.8	18.2	20.6	18.9	22.5
F1ADK9_Aj_FT	35.2	100	67.3	15.6	18.8	19.7	19.1	17.9	22.7	19.2	16.9	16.9	18.4	20.5	19.8	22.1
G7XM46_Ak_FTF	34.6	67.3	100	17.7	20.3	21.3	19.0	18.8	24.1	19.1	16.3	17.2	19.0	18.8	19.1	21.9
Q8A6W6_Bt_Bt1760	14.1	15.6	17.7	100	18.1	17.5	17.5	15.5	18.4	17.8	20.2	18.4	18.2	17.2	17.3	20.6
E3PQS3_Pt_S6FTF	20.8	18.8	20.3	18.1	100	41.1	39.4	24.6	29.9	20.2	22.1	21.0	19.3	23.0	25.3	28.5
Q43866_At_INV1	23.6	19.7	21.3	17.5	41.1	100	50.9	27.9	26.6	22.5	21.8	21.9	22.0	24.0	26.5	29.0
Q93X60_Ci_FF	20.7	19.1	19.0	17.5	39.4	50.9	100	24.1	28.8	21.6	20.5	20.5	22.5	24.2	25.1	28.2
A2TLS9_Bl_FF	19.7	17.9	18.8	15.5	24.6	27.9	24.1	100	28.0	23.7	22.0	23.0	22.3	23.6	23.8	30.5
O33833_Tm_Inv	24.4	22.7	24.1	18.4	29.9	26.6	28.8	28.0	100	24.2	28.1	29.7	26.2	29.0	29.5	33.9
Q9KJD0_Pu_FTF	17.0	19.2	19.1	17.8	20.2	22.5	21.6	23.7	24.2	100	38.6	25.1	23.6	26.5	26.9	29.7
W8GV60_Bl_LevB1	16.7	16.9	16.3	20.2	22.1	21.8	20.5	22.0	28.1	38.6	100	28.1	26.6	25.3	29.7	32.8
P00724_Sc_INV2	18.8	16.9	17.2	18.4	21.0	21.9	20.5	23.0	29.7	25.1	28.1	100	48.2	27.9	32.7	34.3
E5D0X5_So_INV	18.2	18.4	19.0	18.2	19.3	22.0	22.5	22.3	26.2	23.6	26.6	48.2	100	28.9	34.6	36.0
O94220_Af_INU2	20.6	20.5	18.8	17.2	23.0	24.0	24.2	23.6	29.0	26.5	25.3	27.9	28.9	100	34.1	36.4
Q96TU3_Aa_INUE	18.9	19.8	19.1	17.3	25.3	26.5	25.1	23.8	29.5	26.9	29.7	32.7	34.6	34.1	100	41.5
P05656_Bs_SACC	22.5	22.1	21.9	20.6	28.5	29.0	28.2	30.5	33.9	29.7	32.8	34.3	36.0	36.4	41.5	100

Figure S4. Identity matrix of protein sequences. Protein sequences retrieved from UniProt [3] and aligned with Clustal Omega [4] are as follows: β-fructofuranosidase of *Xanthophyllomyces dendrorhous* (UniProt: B8YJM2), fructosyltransferase of *Aspergillus japonicus* (UniProt: F1ADK9), fructosyltransferase of *A. kawachii* (UniProt: G7XM46), endo-levanase of *Bacteroides thetaiotaomicron* (UniProt: Q8A6W6), sucrose:{sucrose/fructan} 6-fructosyltransferase of *Pachysandra terminalis* (UniProt: E3PQS3), cell-wall invertase of *Arabidopsis thaliana* (UniProt: Q43866), fructan 1-exohydrolase of *Cichorium intybus* (UniProt: Q93X60), β-fructofuranosidase of *Bifidobacterium longum* (UniProt: A2TLS9), invertase of *Thermotoga maritima* (UniProt: O33833), levan fructotransferase of *Paenarthrobacter ureafaciens* (UniProt: Q9KJD0), endo-levanase of *Bacillus licheniformis* (UniProt: W8GV60), invertase of *Saccharomyces cerevisiae* (UniProt: P00724), invertase of *Schwanniomyces occidentalis* (UniProt: E5D0X5), endo-inulinase of *A. ficuum* (UniProt: O94220), exo-inulinase of *A. awamori* (UniProt: Q96TU3) and levanase of *B. subtilis* (UniProt: P05656).

Table S5. Protein constructs, plasmids and primers used in this work. Molecular weight (Mw) and pI of the proteins was calculated in the ExPASy Server [5]

**Protein construct**<br>Deleted domain	**Plasmid***	**Primers/comments¤**	**Protein length (aa)/Mw /pI**
**BT1760[#]**	pURI3Cter	FW: 5'-**TAACTTTAAGAAGGAGATATACAT*ATG***GACGAGACTGACCCCATCTTG-3'<br>REV: 5'-**GCTATTAATGATGATGATGATGATG**ATAAGTGCTTACCTGAACGTCTG-3'	508/57.9 kDa;<br>pI: 5.24
**BT1760E221A**	pURI3Cter	Mutation primer: FW 5'-ATCGTTTCTACGCGTGTCCGG-3'	508/57.9 kDa;<br>pI: 5.24
**BT1760$_{1-349}$**<br>C-terminal	pURI3Cter	FW: 5'-**TAACTTTAAGAAGGAGATATACAT*ATG***GACGAGACTGACCCCATCTTG-3'<br>REV: 5'-**GCTA*TTA*ATGATGATGATGATGATG**TGCGGAAGTGTATTTACGGTC-3'	355/40.5 kDa;<br>pI: 5.14
**BT1760$_{1-338}$**<br>C-terminal	pURI3Cter	FW: 5'-**TAACTTTAAGAAGGAGATATACAT*ATG***GACGAGACTGACCCCATCTTG-3'<br>REV: 5'-**GCTA*TTA*ATGATGATGATGATGATG**TACTCCGAGGGTTAAAGTACC-3'	344/39.3 kDa;<br>pI: 5.13
**BT1760$_{340-508}$**<br>N-terminal	pURI3Cter	FW: 5'-**TAACTTTAAGAAGGAGATATACAT*ATG***GCAATCGACCGTAAATACACT-3'<br>REV: 5'-**GCTA*TTA*ATGATGATGATGATGATG**ATAAGTGCTTACCTGAACGTCTG-3'	169/19.3 kDa;<br>pI: 6.7
**BT1760$_{348-508}$**<br>N-terminal	pURI3Cter	FW: 5'-**TAACTTTAAGAAGGAGATATACAT*ATG***GCACAAGAAGTGAAAGTAAT-3'<br>REV: 5'-**GCTA*TTA*ATGATGATGATGATGATG**ATAAGTGCTTACCTGAACGTCTG-3'	169/19.3 kDa;<br>pI: 6.7
**BsCBM66[#]**	pURI3Cter	FW: 5'-**TAACTTTAAGAAGGAGATATACAT*ATG***GGAACGACACCTTTTATGTCC-3'<br>REV: 5'-**GCTA*TTA*ATGATGATGATGATGATG**AGACTCCTTCGTTACATTCTG-3'	171/19.0 kDa;<br>pI: 5.65
**BsCBM66-BT1760**	pET28a	BsCBM66 FW: 5'-ACT*CCATGG*GAACGACACCTTTTATGTCCA-3'<br>REV: 5'-AAA*GGATCC*AGACTCCTTCGTTACATTCTGA-3'<br>BT1760 FW: 5'-GTT*GGATCC*GACGAGACTGACCCCATCTTG-3'<br>REV: 5'-ATA*CTCGAG*ATAAGTGCTTACCTGAACGTC-3'	670/75.6 kDa;<br>pI: 5.11

* Endo-levanase variants in pURI3Cter plasmid were constructed as in [1,6] and pET28a plasmid was obtained from Merck, Germany.

¤ Bold font in primer sequences highlights the nucleotides annealing with the pURI3Cter vector, start and stop codons are in italics. In BT1760E221A, the triplet introducing a mutation is underlined. In the case of BsCBM66-BT1760 construct, primers containing restriction site (underlined, italics) were used to amplify the specific DNA segment: the primers for BsCBM66 amplification contained restriction sites (*Nco*I and *Bam*HI) at the ends, the primers for BT1760 had *Bam*HI and *Xho*I sites. The two amplified fragments were merged *via* *Bam*HI restriction site. The pET28a vector was used for cloning resulting in pET28-BsCBM66-BT1760.

The plasmid carrying the *BT1760* gene was constructed as in [1] and *Bacillus subtilis* 168 DSM 23778 was purchased from DSMZ (Germany). Genomic DNA of the bacterium was extracted with PowerSoil DNA Isolation Kit (MO BIO, USA).

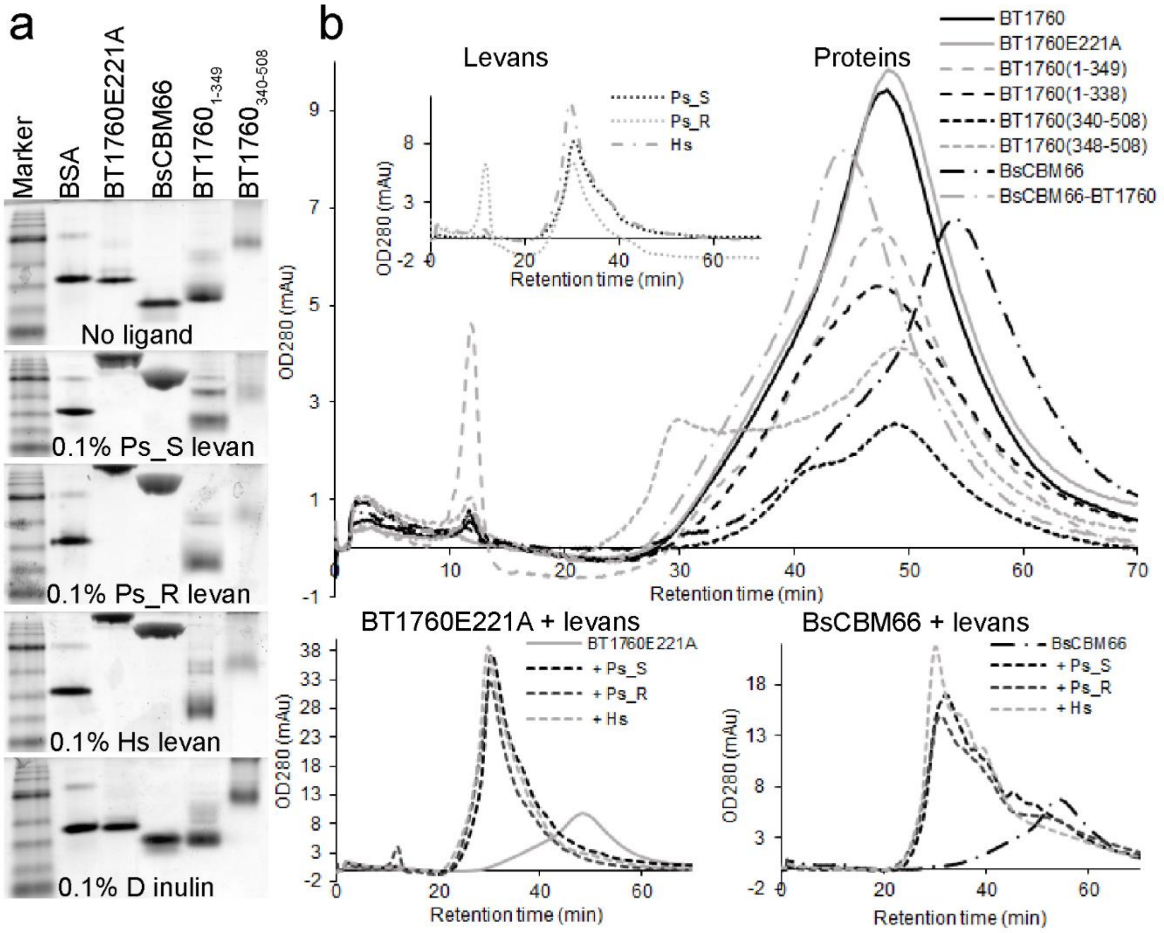

Figure S5. Polyfructan binding to endolevanase variants. (a) Affinity gel electrophoresis was used to assay the binding of proteins to polyfructans. Continuous native polyacrylamide gels consisting of 12.5% (*w/v*) acrylamide in 25 mM Tris/250 mM glycine buffer (pH 8.3) were prepared as in [7]. Three different types of native gels were used: 1) no fructan added (no ligand), 2) levan (0.1% Ps_S levan, 0.1% Ps-R levan, 0.1% Hs levan designated in Materials and Methods in the main text) or 3) dahlia inulin (0.1% D inulin) added to the gel prior to polymerization. Purified proteins and bovine serum albumin (BSA from Pierce™ BCA Protein Assay Kit, Thermo Scientific, USA) used as a noninteracting negative control; 2 µg each, were loaded onto the gels. Proteins were visualized by staining of gels with Coomassie Blue. Panel (a) is composed of cropped gels from separate experiments and aligned according to the protein size marker (Naxo, Estonia) with two prominent bands corresponding to M_w of 75 and 25 kDa. The images were not digitally modified except for applying grayscale mode, the originals are presented in Supplementary Figure S5.

(b) Econo-Column® Chromatography Columns, 1.0 × 30 cm (Bio-Rad, USA) was used for analytical size-exclusion chromatography of proteins. The column was packed with Sephacryl S-200 HR resin (GE Healthcare, USA) attached to the ÄKTAprime plus system and equilibrated with 50 mM Na-phosphate buffer (pH 7.0) supplemented with 150 mM NaCl. Flow rate was adjusted to 0.3 mL/min and the sample size of 500 µL was transferred to the column. In total 200 µg of purified protein and/or 500 µg of fructan was loaded to the column in the same buffer. In size-exclusion column smaller molecules invade the porous matrix and elute from the column later [8,9].

Table S6. Kinetic parameters of wild-type endo-levanase BT1760 and of CBM66-amended variant BsCBM66-BT1760

**BT1760***	**k_{cat}** (1/s)	**K_M** (g/L)	**k_{cat}/K_M** (1/sec x g/L)
Ps_S	**466.8**±27.7	**13.6**±1.5	**34.3**
Ps_R	**366.7**±23.3	**12.2**±1.5	**30.1**
Hs	**333.0**±26.4	**7.8**±1.3	**42.7**
**BsCBM66-BT1760**			
Ps_S	**389.7**±21.2	**10.9**±1.1	**35.8**
Ps_R	**374.5**±40.7	**12.3**±2.3	**30.4**
Hs	**374.7**±25.6	**7.4**±1.1	**50.6**

*Data from [1]

Kinetic parameters of levan degradation by endo-levanases (BT1760 or BsCBM66-BT1760) were calculated on three levans by recording specific activities at varied substrate concentrations. The data were analysed using Sigma Plot 2001 Enzyme Kinetic Module (Systat Software Inc., USA). Mean values ± standard deviation of at least two independent measurements are presented

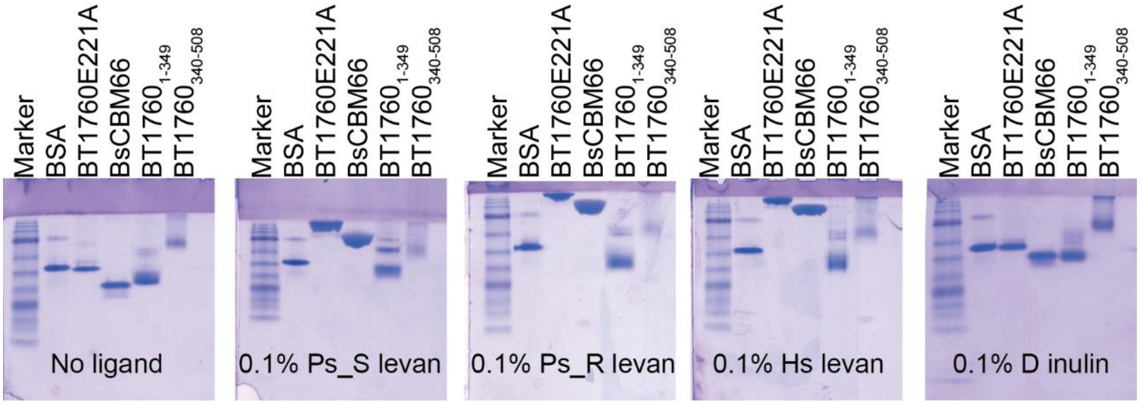

Figure S6. Original images of gels from affinity gel electrophoresis presented in Figure S5 (a).

References:

1. Mardo, K. *et al.* A highly active endo-levanase BT1760 of a dominant mammalian gut commensal Bacteroides thetaiotaomicron cleaves not only various bacterial levans, but also levan of timothy grass. *PLoS One* **12**, e0169989 (2017).
2. Holm, L. & Laakso, L. M. Dali server update. *Nucleic Acids Res.* **44**, W351–W355 (2016).
3. UniProt Consortium, T. UniProt: the universal protein knowledgebase. *Nucleic Acids Res.* **46**, 2699 (2018).
4. Sievers, F. *et al.* Fast, scalable generation of high-quality protein multiple sequence alignments using Clustal Omega. *Mol. Syst. Biol.* **7**, 539 (2011).
5. Gasteiger, E. *et al.* Protein Identification and Analysis Tools on the ExPASy Server. in *The Proteomics Protocols Handbook, (2005).* (ed. Walker, J. M.) 571–607 (Humana Press, 2005). doi:10.1385/1592598900
6. Curiel, J. A., de Las Rivas, B., Mancheño, J. M. & Muñoz, R. The pURI family of expression vectors: A versatile set of ligation independent cloning plasmids for producing recombinant His-fusion proteins. *Protein Expr. Purif.* **76**, 44–53 (2011).
7. Xie, H. *et al.* Role of hydrogen bonding in the interaction between a xylan binding module and xylan. *Biochemistry* **40**, 5700–5707 (2001).
8. Jonker, N., Kool, J., Irth, H. & Niessen, W. M. A. Recent developments in protein-ligand affinity mass spectrometry. *Anal. Bioanal. Chem.* **399**, 2669–2681 (2011).
9. Tao, Y. & Zhang, L. Characterization of polysaccharide-protein complexes by size-exclusion chromatography combined with three detectors. *Carbohydr. Res.* **343**, 2251–2257 (2008).
